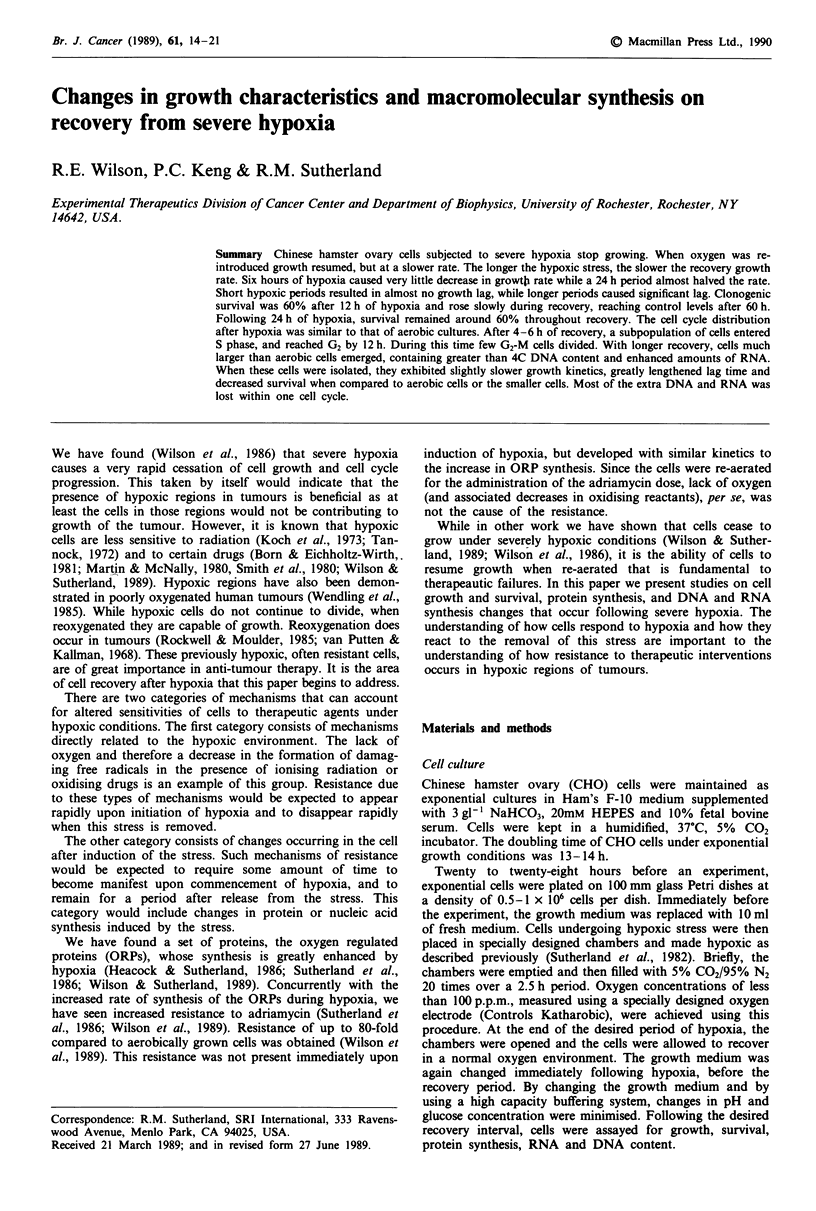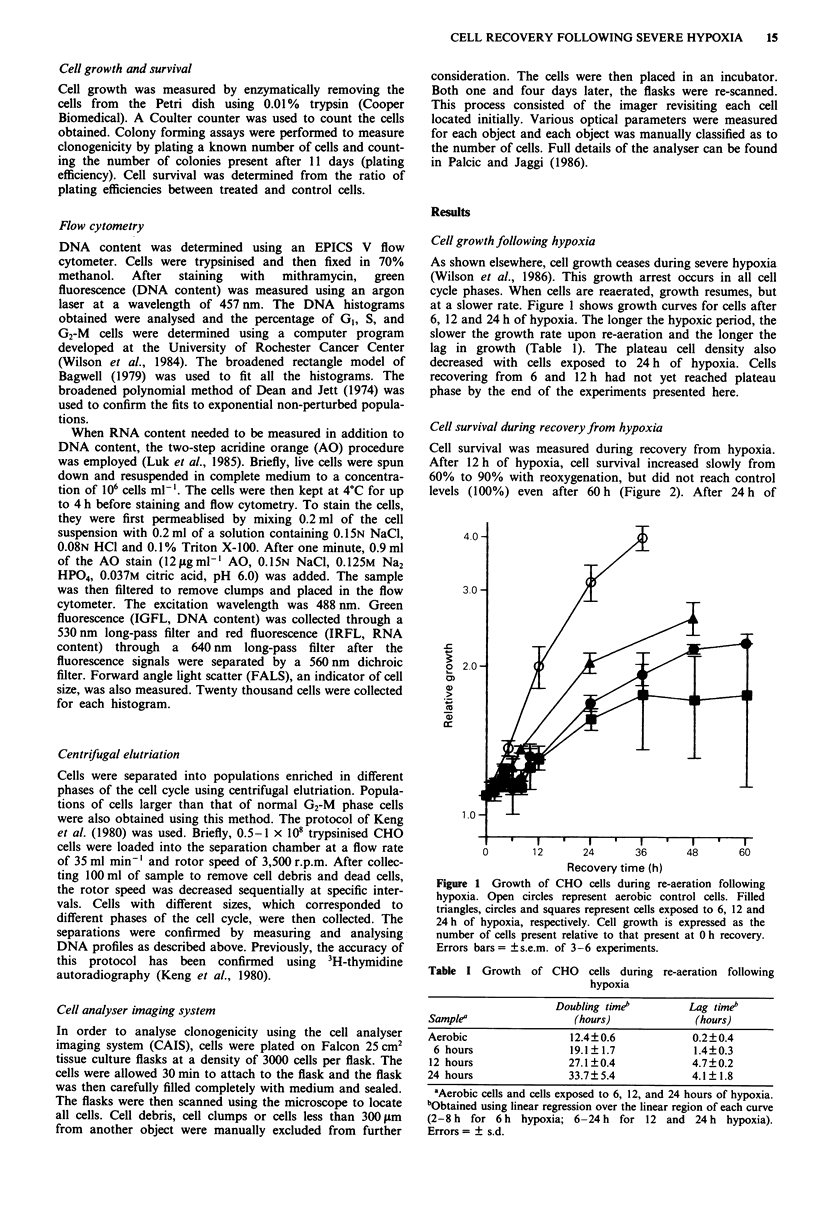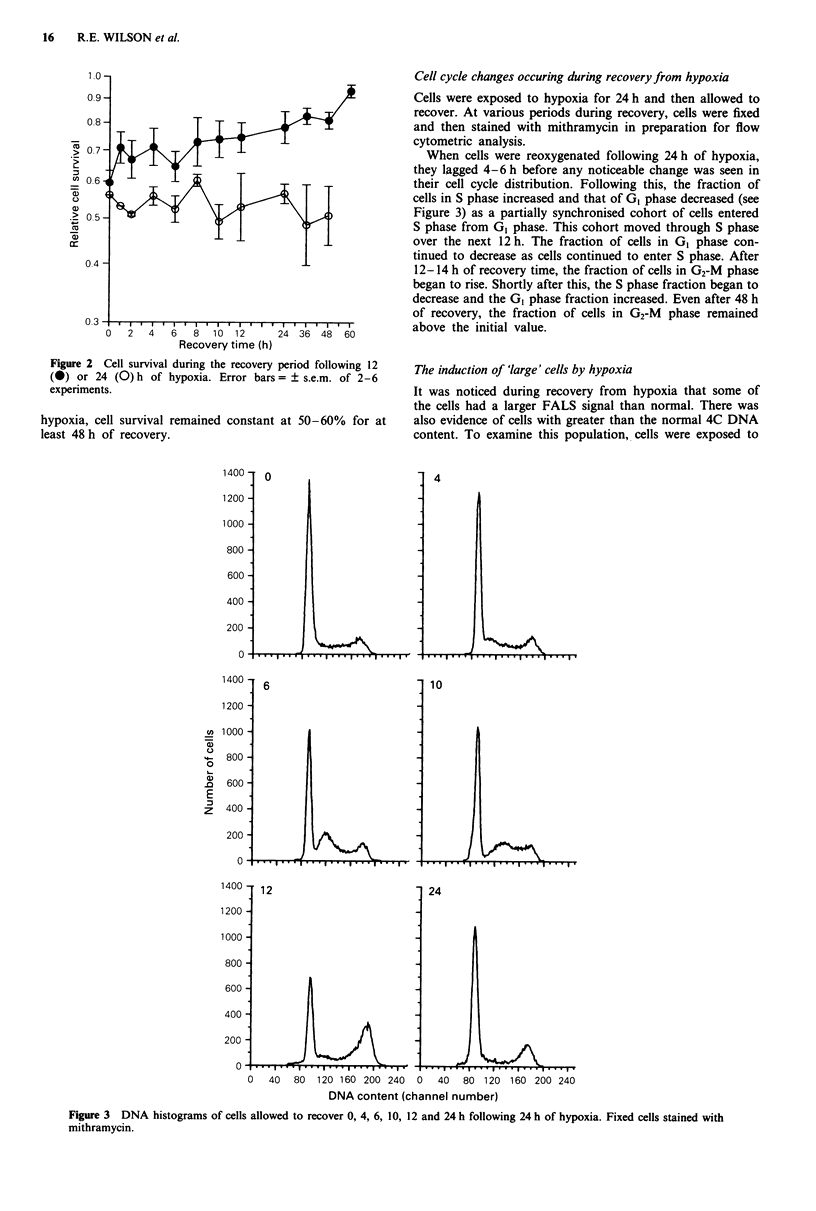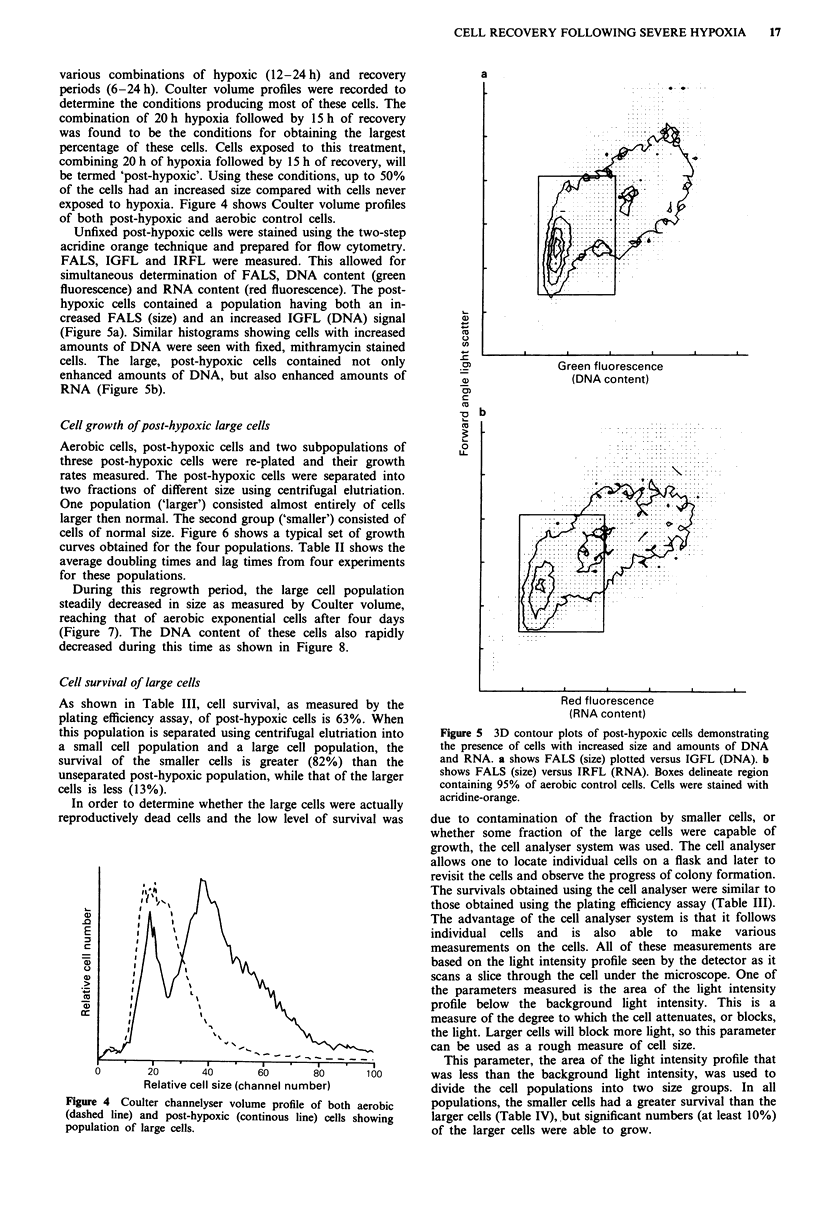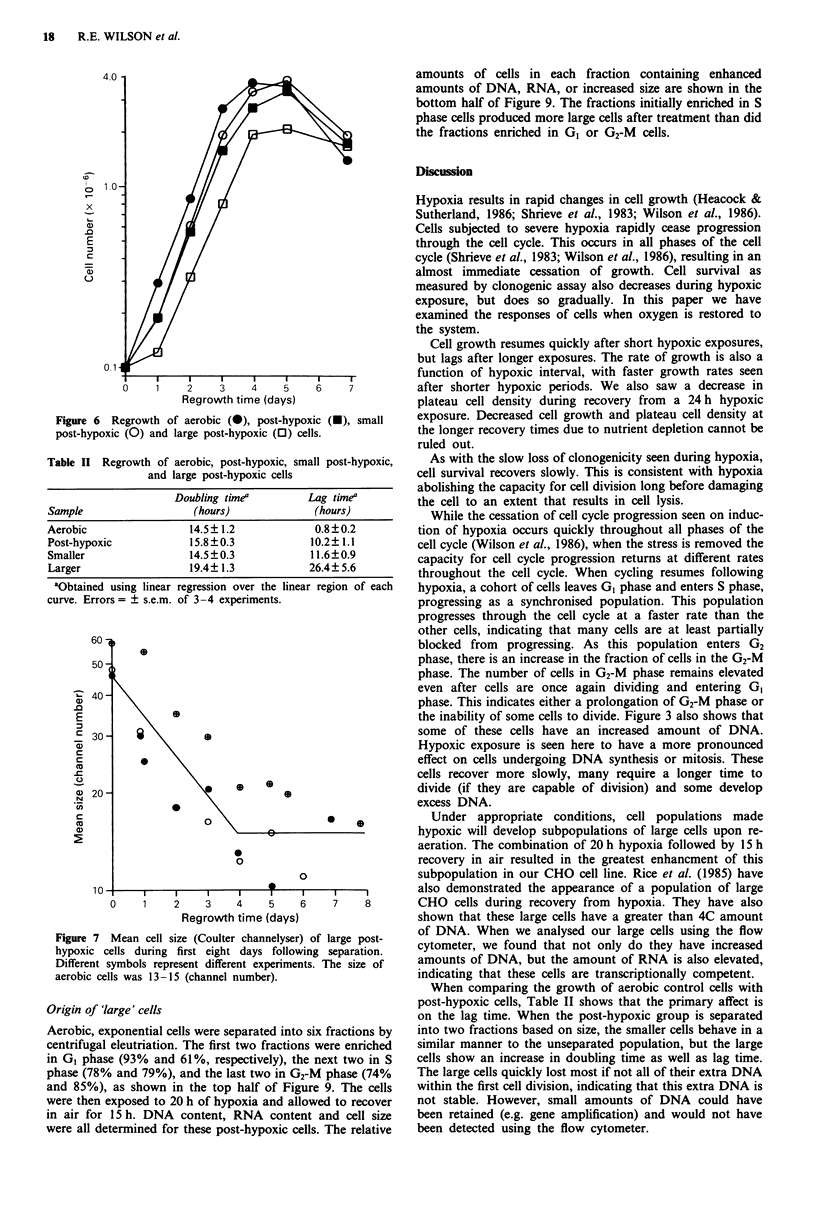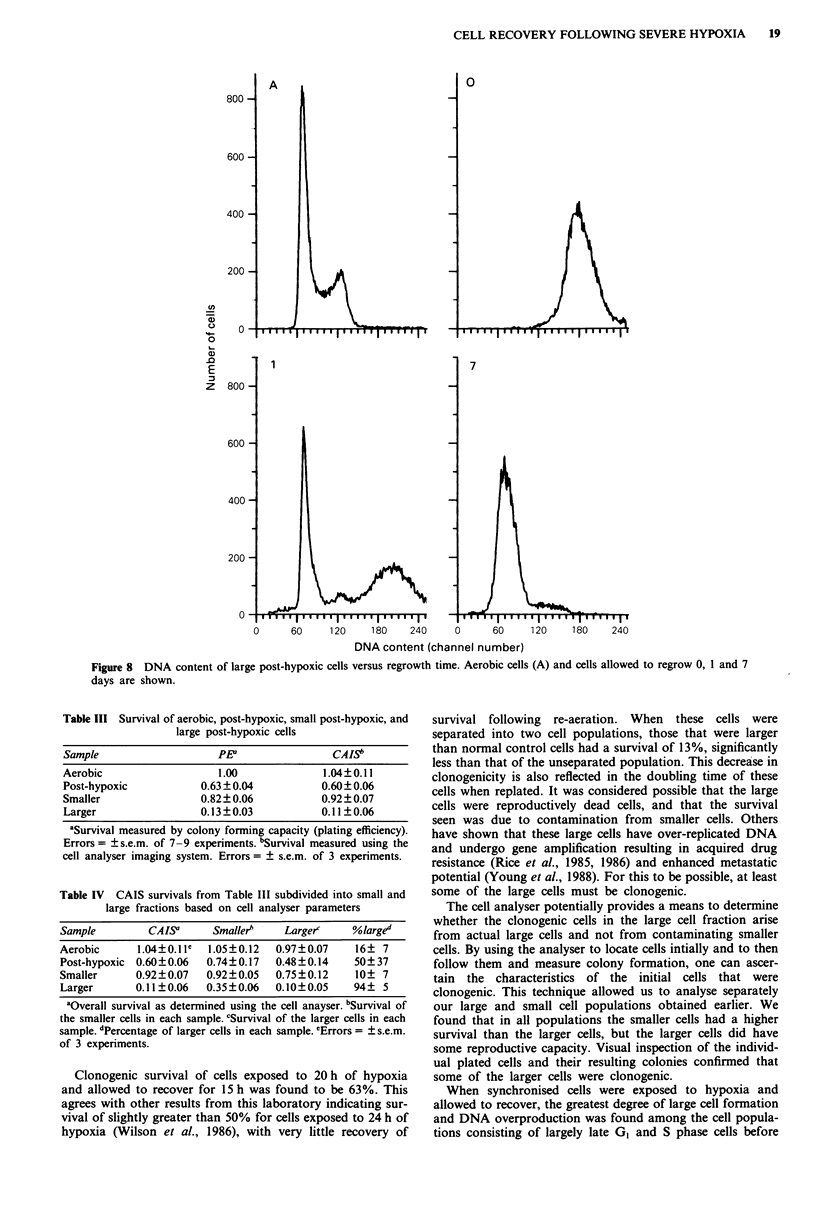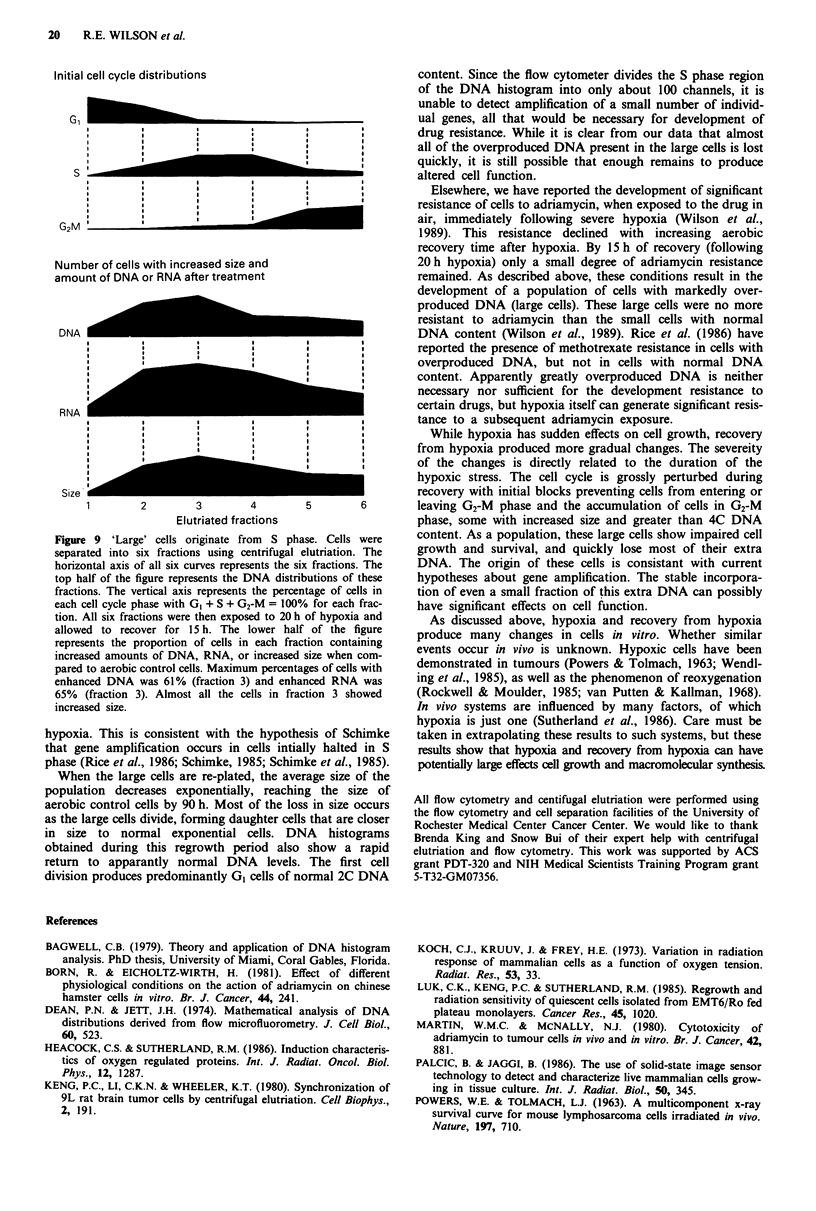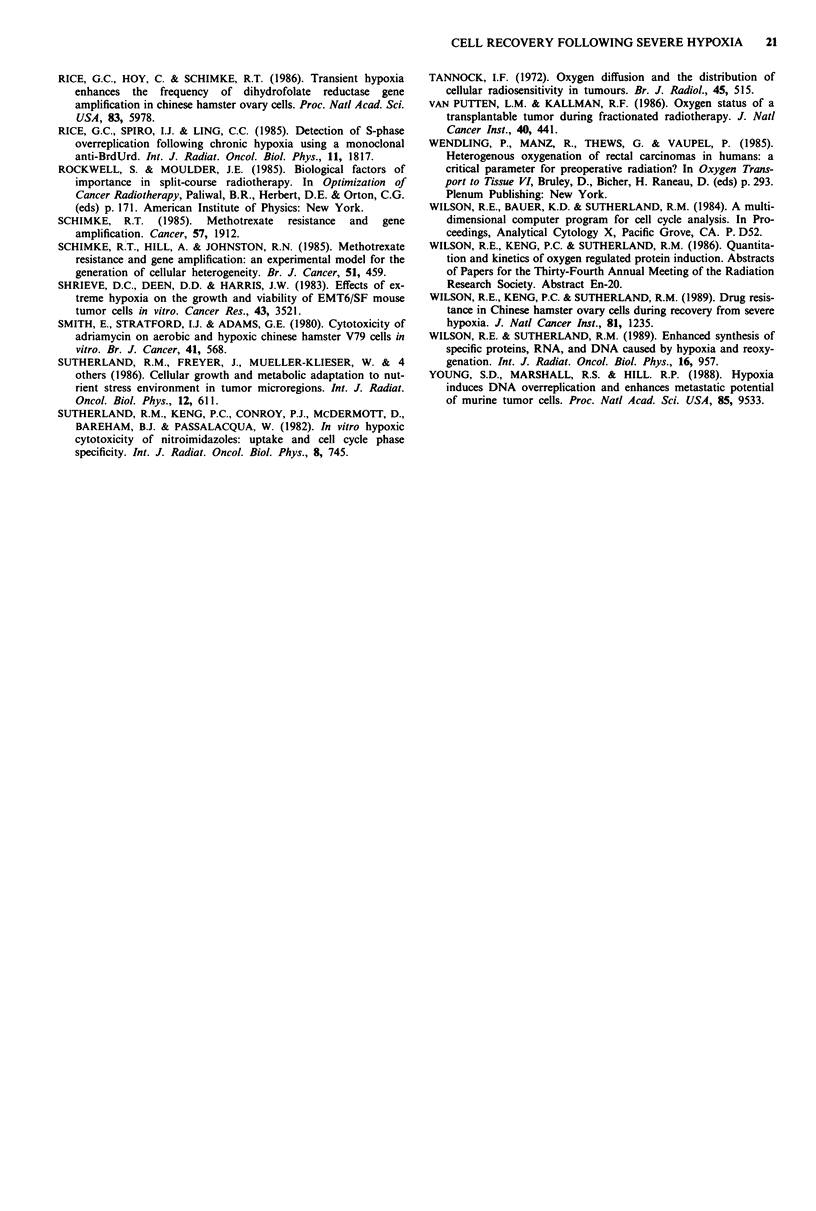# Changes in growth characteristics and macromolecular synthesis on recovery from severe hypoxia.

**DOI:** 10.1038/bjc.1990.5

**Published:** 1990-01

**Authors:** R. E. Wilson, P. C. Keng, R. M. Sutherland

**Affiliations:** Experimental Therapeutics Division of Cancer Center, University of Rochester, NY 14642.

## Abstract

Chinese hamster ovary cells subjected to severe hypoxia stop growing. When oxygen was reintroduced growth resumed, but at a slower rate. The longer the hypoxic stress, the slower the recovery growth rate. Six hours of hypoxia caused very little decrease in growth rate while a 24 h period almost halved the rate. Short hypoxic periods resulted in almost no growth lag, while longer periods caused significant lag. Clonogenic survival was 60% after 12 h of hypoxia and rose slowly during recovery, reaching control levels after 60 h. Following 24 h of hypoxia, survival remained around 60% throughout recovery. The cell cycle distribution after hypoxia was similar to that of aerobic cultures. After 4-6 h of recovery, a subpopulation of cells entered S phase, and reached G2 by 12 h. During this time few G2-M cells divided. With longer recovery, cells much larger than aerobic cells emerged, containing greater than 4C DNA content and enhanced amounts of RNA. When these cells were isolated, they exhibited slightly slower growth kinetics, greatly lengthened lag time and decreased survival when compared to aerobic cells or the smaller cells. Most of the extra DNA and RNA was lost within one cell cycle.


Br. J. Cancer (1989), 61, 14–21 © Macmillan Press Ltd., 1990

### Changes in growth characteristics and macromolecular synthesis on recovery from severe hypoxia

R.E. Wilson, P.C. Keng & R.M. Sutherland

*Experimental Therapeutics Division of Cancer Center and Department of Biophysics, University of Rochester, Rochester, NY 14642, USA.*

**Summary** Chinese hamster ovary cells subjected to severe hypoxia stop growing. When oxygen was re-introduced growth resumed, but at a slower rate. The longer the hypoxic stress, the slower the recovery growth rate. Six hours of hypoxia caused very little decrease in growth rate while a 24 h period almost halved the rate. Short hypoxic periods resulted in almost no growth lag, while longer periods caused significant lag. Clonogenic survival was 60% after 12 h of hypoxia and rose slowly during recovery, reaching control levels after 60 h. Following 24 h of hypoxia, survival remained around 60% throughout recovery. The cell cycle distribution after hypoxia was similar to that of aerobic cultures. After 4–6 h of recovery, a subpopulation of cells entered S phase, and reached $G_2$ by 12 h. During this time few $G_2$-M cells divided. With longer recovery, cells much larger than aerobic cells emerged, containing greater than 4C DNA content and enhanced amounts of RNA. When these cells were isolated, they exhibited slightly slower growth kinetics, greatly lengthened lag time and decreased survival when compared to aerobic cells or the smaller cells. Most of the extra DNA and RNA was lost within one cell cycle.

We have found (Wilson *et al.*, 1986) that severe hypoxia causes a very rapid cessation of cell growth and cell cycle progression. This taken by itself would indicate that the presence of hypoxic regions in tumours is beneficial as at least the cells in those regions would not be contributing to growth of the tumour. However, it is known that hypoxic cells are less sensitive to radiation (Koch *et al.*, 1973; Tannock, 1972) and to certain drugs (Born & Eichholtz-Wirth, 1981; Martin & McNally, 1980, Smith *et al.*, 1980; Wilson & Sutherland, 1989). Hypoxic regions have also been demonstrated in poorly oxygenated human tumours (Wendling *et al.*, 1985). While hypoxic cells do not continue to divide, when reoxygenated they are capable of growth. Reoxygenation does occur in tumours (Rockwell & Moulder, 1985; van Putten & Kallman, 1968). These previously hypoxic, often resistant cells, are of great importance in anti-tumour therapy. It is the area of cell recovery after hypoxia that this paper begins to address.

There are two categories of mechanisms that can account for altered sensitivities of cells to therapeutic agents under hypoxic conditions. The first category consists of mechanisms directly related to the hypoxic environment. The lack of oxygen and therefore a decrease in the formation of damaging free radicals in the presence of ionising radiation or oxidising drugs is an example of this group. Resistance due to these types of mechanisms would be expected to appear rapidly upon initiation of hypoxia and to disappear rapidly when this stress is removed.

The other category consists of changes occurring in the cell after induction of the stress. Such mechanisms of resistance would be expected to require some amount of time to become manifest upon commencement of hypoxia, and to remain for a period after release from the stress. This category would include changes in protein or nucleic acid synthesis induced by the stress.

We have found a set of proteins, the oxygen regulated proteins (ORPs), whose synthesis is greatly enhanced by hypoxia (Heacock & Sutherland, 1986; Sutherland *et al.*, 1986; Wilson & Sutherland, 1989). Concurrently with the increased rate of synthesis of the ORPs during hypoxia, we have seen increased resistance to adriamycin (Sutherland *et al.*, 1986; Wilson *et al.*, 1989). Resistance of up to 80-fold compared to aerobically grown cells was obtained (Wilson *et al.*, 1989). This resistance was not present immediately upon induction of hypoxia, but developed with similar kinetics to the increase in ORP synthesis. Since the cells were re-aerated for the administration of the adriamycin dose, lack of oxygen (and associated decreases in oxidising reactants), *per se*, was not the cause of the resistance.

While in other work we have shown that cells cease to grow under severely hypoxic conditions (Wilson & Sutherland, 1989; Wilson *et al.*, 1986), it is the ability of cells to resume growth when re-aerated that is fundamental to therapeautic failures. In this paper we present studies on cell growth and survival, protein synthesis, and DNA and RNA synthesis changes that occur following severe hypoxia. The understanding of how cells respond to hypoxia and how they react to the removal of this stress are important to the understanding of how resistance to therapeutic interventions occurs in hypoxic regions of tumours.

Correspondence: R.M. Sutherland, SRI International, 333 Ravenswood Avenue, Menlo Park, CA 94025, USA.
Received 21 March 1989; and in revised form 27 June 1989.

### Materials and methods

### *Cell culture*

Chinese hamster ovary (CHO) cells were maintained as exponential cultures in Ham's F-10 medium supplemented with 3 $gl^{-1}$ $NaHCO_3$, 20mM HEPES and 10% fetal bovine serum. Cells were kept in a humidified, 37°C, 5% $CO_2$ incubator. The doubling time of CHO cells under exponential growth conditions was 13–14 h.

Twenty to twenty-eight hours before an experiment, exponential cells were plated on 100 mm glass Petri dishes at a density of $0.5-1 \times 10^6$ cells per dish. Immediately before the experiment, the growth medium was replaced with 10 ml of fresh medium. Cells undergoing hypoxic stress were then placed in specially designed chambers and made hypoxic as described previously (Sutherland *et al.*, 1982). Briefly, the chambers were emptied and then filled with 5% $CO_2$/95% $N_2$ 20 times over a 2.5 h period. Oxygen concentrations of less than 100 p.p.m., measured using a specially designed oxygen electrode (Controls Katharobic), were achieved using this procedure. At the end of the desired period of hypoxia, the chambers were opened and the cells were allowed to recover in a normal oxygen environment. The growth medium was again changed immediately following hypoxia, before the recovery period. By changing the growth medium and by using a high capacity buffering system, changes in pH and glucose concentration were minimised. Following the desired recovery interval, cells were assayed for growth, survival, protein synthesis, RNA and DNA content.

*Cell growth and survival*

Cell growth was measured by enzymatically removing the cells from the Petri dish using 0.01% trypsin (Cooper Biomedical). A Coulter counter was used to count the cells obtained. Colony forming assays were performed to measure clonogenicity by plating a known number of cells and counting the number of colonies present after 11 days (plating efficiency). Cell survival was determined from the ratio of plating efficiencies between treated and control cells.

*Flow cytometry*

DNA content was determined using an EPICS V flow cytometer. Cells were trypsinised and then fixed in 70% methanol. After staining with mithramycin, green fluorescence (DNA content) was measured using an argon laser at a wavelength of 457 nm. The DNA histograms obtained were analysed and the percentage of $G_1$, S, and $G_2$-M cells were determined using a computer program developed at the University of Rochester Cancer Center (Wilson *et al.*, 1984). The broadened rectangle model of Bagwell (1979) was used to fit all the histograms. The broadened polynomial method of Dean and Jett (1974) was used to confirm the fits to exponential non-perturbed populations.

When RNA content needed to be measured in addition to DNA content, the two-step acridine orange (AO) procedure was employed (Luk *et al.*, 1985). Briefly, live cells were spun down and resuspended in complete medium to a concentration of $10^6$ cells $ml^{-1}$. The cells were then kept at 4°C for up to 4 h before staining and flow cytometry. To stain the cells, they were first permeablised by mixing 0.2 ml of the cell suspension with 0.2 ml of a solution containing 0.15N NaCl, 0.08N HCl and 0.1% Triton X-100. After one minute, 0.9 ml of the AO stain (12 μg $ml^{-1}$ AO, 0.15N NaCl, 0.125M $Na_2HPO_4$, 0.037M citric acid, pH 6.0) was added. The sample was then filtered to remove clumps and placed in the flow cytometer. The excitation wavelength was 488 nm. Green fluorescence (IGFL, DNA content) was collected through a 530 nm long-pass filter and red fluorescence (IRFL, RNA content) through a 640 nm long-pass filter after the fluorescence signals were separated by a 560 nm dichroic filter. Forward angle light scatter (FALS), an indicator of cell size, was also measured. Twenty thousand cells were collected for each histogram.

*Centrifugal elutriation*

Cells were separated into populations enriched in different phases of the cell cycle using centrifugal elutriation. Populations of cells larger than that of normal $G_2$-M phase cells were also obtained using this method. The protocol of Keng *et al.* (1980) was used. Briefly, 0.5–1 × $10^8$ trypsinised CHO cells were loaded into the separation chamber at a flow rate of 35 ml $min^{-1}$ and rotor speed of 3,500 r.p.m. After collecting 100 ml of sample to remove cell debris and dead cells, the rotor speed was decreased sequentially at specific intervals. Cells with different sizes, which corresponded to different phases of the cell cycle, were then collected. The separations were confirmed by measuring and analysing DNA profiles as described above. Previously, the accuracy of this protocol has been confirmed using $^3$H-thymidine autoradiography (Keng *et al.*, 1980).

*Cell analyser imaging system*

In order to analyse clonogenicity using the cell analyser imaging system (CAIS), cells were plated on Falcon 25 $cm^2$ tissue culture flasks at a density of 3000 cells per flask. The cells were allowed 30 min to attach to the flask and the flask was then carefully filled completely with medium and sealed. The flasks were then scanned using the microscope to locate all cells. Cell debris, cell clumps or cells less than 300 μm from another object were manually excluded from further consideration. The cells were then placed in an incubator. Both one and four days later, the flasks were re-scanned. This process consisted of the imager revisiting each cell located initially. Various optical parameters were measured for each object and each object was manually classified as to the number of cells. Full details of the analyser can be found in Palcic and Jaggi (1986).

### Results

*Cell growth following hypoxia*

As shown elsewhere, cell growth ceases during severe hypoxia (Wilson *et al.*, 1986). This growth arrest occurs in all cell cycle phases. When cells are reaerated, growth resumes, but at a slower rate. Figure 1 shows growth curves for cells after 6, 12 and 24 h of hypoxia. The longer the hypoxic period, the slower the growth rate upon re-aeration and the longer the lag in growth (Table 1). The plateau cell density also decreased with cells exposed to 24 h of hypoxia. Cells recovering from 6 and 12 h had not yet reached plateau phase by the end of the experiments presented here.

*Cell survival during recovery from hypoxia*

Cell survival was measured during recovery from hypoxia. After 12 h of hypoxia, cell survival increased slowly from 60% to 90% with reoxygenation, but did not reach control levels (100%) even after 60 h (Figure 2). After 24 h of

**Figure 1** Growth of CHO cells during re-aeration following hypoxia. Open circles represent aerobic control cells. Filled triangles, circles and squares represent cells exposed to 6, 12 and 24 h of hypoxia, respectively. Cell growth is expressed as the number of cells present relative to that present at 0 h recovery. Errors bars = ± s.e.m. of 3–6 experiments.

**Table 1** Growth of CHO cells during re-aeration following hypoxia

| *Sample*[a] | *Doubling time*[b] *(hours)* | *Lag time*[b] *(hours)* |
|---|---|---|
| Aerobic | 12.4 ± 0.6 | 0.2 ± 0.4 |
| 6 hours | 19.1 ± 1.7 | 1.4 ± 0.3 |
| 12 hours | 27.1 ± 0.4 | 4.7 ± 0.2 |
| 24 hours | 33.7 ± 5.4 | 4.1 ± 1.8 |

[a]Aerobic cells and cells exposed to 6, 12, and 24 hours of hypoxia.
[b]Obtained using linear regression over the linear region of each curve (2–8 h for 6 h hypoxia; 6–24 h for 12 and 24 h hypoxia). Errors = ± s.d.

**Figure 2** Cell survival during the recovery period following 12 (●) or 24 (○) h of hypoxia. Error bars = ± s.e.m. of 2–6 experiments.

hypoxia, cell survival remained constant at 50–60% for at least 48 h of recovery.

*Cell cycle changes occuring during recovery from hypoxia*

Cells were exposed to hypoxia for 24 h and then allowed to recover. At various periods during recovery, cells were fixed and then stained with mithramycin in preparation for flow cytometric analysis.

When cells were reoxygenated following 24 h of hypoxia, they lagged 4–6 h before any noticeable change was seen in their cell cycle distribution. Following this, the fraction of cells in S phase increased and that of $G_1$ phase decreased (see Figure 3) as a partially synchronised cohort of cells entered S phase from $G_1$ phase. This cohort moved through S phase over the next 12 h. The fraction of cells in $G_1$ phase continued to decrease as cells continued to enter S phase. After 12–14 h of recovery time, the fraction of cells in $G_2$-M phase began to rise. Shortly after this, the S phase fraction began to decrease and the $G_1$ phase fraction increased. Even after 48 h of recovery, the fraction of cells in $G_2$-M phase remained above the initial value.

*The induction of 'large' cells by hypoxia*

It was noticed during recovery from hypoxia that some of the cells had a larger FALS signal than normal. There was also evidence of cells with greater than the normal 4C DNA content. To examine this population, cells were exposed to

**Figure 3** DNA histograms of cells allowed to recover 0, 4, 6, 10, 12 and 24 h following 24 h of hypoxia. Fixed cells stained with mithramycin.

various combinations of hypoxic (12–24 h) and recovery periods (6–24 h). Coulter volume profiles were recorded to determine the conditions producing most of these cells. The combination of 20 h hypoxia followed by 15 h of recovery was found to be the conditions for obtaining the largest percentage of these cells. Cells exposed to this treatment, combining 20 h of hypoxia followed by 15 h of recovery, will be termed 'post-hypoxic'. Using these conditions, up to 50% of the cells had an increased size compared with cells never exposed to hypoxia. Figure 4 shows Coulter volume profiles of both post-hypoxic and aerobic control cells.

Unfixed post-hypoxic cells were stained using the two-step acridine orange technique and prepared for flow cytometry. FALS, IGFL and IRFL were measured. This allowed for simultaneous determination of FALS, DNA content (green fluorescence) and RNA content (red fluorescence). The post-hypoxic cells contained a population having both an increased FALS (size) and an increased IGFL (DNA) signal (Figure 5a). Similar histograms showing cells with increased amounts of DNA were seen with fixed, mithramycin stained cells. The large, post-hypoxic cells contained not only enhanced amounts of DNA, but also enhanced amounts of RNA (Figure 5b).

*Cell growth of post-hypoxic large cells*

Aerobic cells, post-hypoxic cells and two subpopulations of threse post-hypoxic cells were re-plated and their growth rates measured. The post-hypoxic cells were separated into two fractions of different size using centrifugal elutriation. One population ('larger') consisted almost entirely of cells larger then normal. The second group ('smaller') consisted of cells of normal size. Figure 6 shows a typical set of growth curves obtained for the four populations. Table II shows the average doubling times and lag times from four experiments for these populations.

During this regrowth period, the large cell population steadily decreased in size as measured by Coulter volume, reaching that of aerobic exponential cells after four days (Figure 7). The DNA content of these cells also rapidly decreased during this time as shown in Figure 8.

*Cell survival of large cells*

As shown in Table III, cell survival, as measured by the plating efficiency assay, of post-hypoxic cells is 63%. When this population is separated using centrifugal elutriation into a small cell population and a large cell population, the survival of the smaller cells is greater (82%) than the unseparated post-hypoxic population, while that of the larger cells is less (13%).

In order to determine whether the large cells were actually reproductively dead cells and the low level of survival was

**Figure 4** Coulter channelyser volume profile of both aerobic (dashed line) and post-hypoxic (continous line) cells showing population of large cells.

**Figure 5** 3D contour plots of post-hypoxic cells demonstrating the presence of cells with increased size and amounts of DNA and RNA. **a** shows FALS (size) plotted versus IGFL (DNA). **b** shows FALS (size) versus IRFL (RNA). Boxes delineate region containing 95% of aerobic control cells. Cells were stained with acridine-orange.

due to contamination of the fraction by smaller cells, or whether some fraction of the large cells were capable of growth, the cell analyser system was used. The cell analyser allows one to locate individual cells on a flask and later to revisit the cells and observe the progress of colony formation. The survivals obtained using the cell analyser were similar to those obtained using the plating efficiency assay (Table III). The advantage of the cell analyser system is that it follows individual cells and is also able to make various measurements on the cells. All of these measurements are based on the light intensity profile seen by the detector as it scans a slice through the cell under the microscope. One of the parameters measured is the area of the light intensity profile below the background light intensity. This is a measure of the degree to which the cell attenuates, or blocks, the light. Larger cells will block more light, so this parameter can be used as a rough measure of cell size.

This parameter, the area of the light intensity profile that was less than the background light intensity, was used to divide the cell populations into two size groups. In all populations, the smaller cells had a greater survival than the larger cells (Table IV), but significant numbers (at least 10%) of the larger cells were able to grow.

**Figure 6** Regrowth of aerobic (●), post-hypoxic (■), small post-hypoxic (○) and large post-hypoxic (□) cells.

**Table II** Regrowth of aerobic, post-hypoxic, small post-hypoxic, and large post-hypoxic cells

| *Sample* | *Doubling time*[a] *(hours)* | *Lag time*[a] *(hours)* |
|---|---|---|
| Aerobic | 14.5±1.2 | 0.8±0.2 |
| Post-hypoxic | 15.8±0.3 | 10.2±1.1 |
| Smaller | 14.5±0.3 | 11.6±0.9 |
| Larger | 19.4±1.3 | 26.4±5.6 |

[a]Obtained using linear regression over the linear region of each curve. Errors = ± s.e.m. of 3–4 experiments.

**Figure 7** Mean cell size (Coulter channelyser) of large post-hypoxic cells during first eight days following separation. Different symbols represent different experiments. The size of aerobic cells was 13–15 (channel number).

### *Origin of 'large' cells*

Aerobic, exponential cells were separated into six fractions by centrifugal eleutriation. The first two fractions were enriched in $G_1$ phase (93% and 61%, respectively), the next two in S phase (78% and 79%), and the last two in $G_2$-M phase (74% and 85%), as shown in the top half of Figure 9. The cells were then exposed to 20 h of hypoxia and allowed to recover in air for 15 h. DNA content, RNA content and cell size were all determined for these post-hypoxic cells. The relative amounts of cells in each fraction containing enhanced amounts of DNA, RNA, or increased size are shown in the bottom half of Figure 9. The fractions initially enriched in S phase cells produced more large cells after treatment than did the fractions enriched in $G_1$ or $G_2$-M cells.

### Discussion

Hypoxia results in rapid changes in cell growth (Heacock & Sutherland, 1986; Shrieve *et al.*, 1983; Wilson *et al.*, 1986). Cells subjected to severe hypoxia rapidly cease progression through the cell cycle. This occurs in all phases of the cell cycle (Shrieve *et al.*, 1983; Wilson *et al.*, 1986), resulting in an almost immediate cessation of growth. Cell survival as measured by clonogenic assay also decreases during hypoxic exposure, but does so gradually. In this paper we have examined the responses of cells when oxygen is restored to the system.

Cell growth resumes quickly after short hypoxic exposures, but lags after longer exposures. The rate of growth is also a function of hypoxic interval, with faster growth rates seen after shorter hypoxic periods. We also saw a decrease in plateau cell density during recovery from a 24 h hypoxic exposure. Decreased cell growth and plateau cell density at the longer recovery times due to nutrient depletion cannot be ruled out.

As with the slow loss of clonogenicity seen during hypoxia, cell survival recovers slowly. This is consistent with hypoxia abolishing the capacity for cell division long before damaging the cell to an extent that results in cell lysis.

While the cessation of cell cycle progression seen on induction of hypoxia occurs quickly throughout all phases of the cell cycle (Wilson *et al.*, 1986), when the stress is removed the capacity for cell cycle progression returns at different rates throughout the cell cycle. When cycling resumes following hypoxia, a cohort of cells leaves $G_1$ phase and enters S phase, progressing as a synchronised population. This population progresses through the cell cycle at a faster rate than the other cells, indicating that many cells are at least partially blocked from progressing. As this population enters $G_2$ phase, there is an increase in the fraction of cells in the $G_2$-M phase. The number of cells in $G_2$-M phase remains elevated even after cells are once again dividing and entering $G_1$ phase. This indicates either a prolongation of $G_2$-M phase or the inability of some cells to divide. Figure 3 also shows that some of these cells have an increased amount of DNA. Hypoxic exposure is seen here to have a more pronounced effect on cells undergoing DNA synthesis or mitosis. These cells recover more slowly, many require a longer time to divide (if they are capable of division) and some develop excess DNA.

Under appropriate conditions, cell populations made hypoxic will develop subpopulations of large cells upon re-aeration. The combination of 20 h hypoxia followed by 15 h recovery in air resulted in the greatest enhancment of this subpopulation in our CHO cell line. Rice *et al.* (1985) have also demonstrated the appearance of a population of large CHO cells during recovery from hypoxia. They have also shown that these large cells have a greater than 4C amount of DNA. When we analysed our large cells using the flow cytometer, we found that not only do they have increased amounts of DNA, but the amount of RNA is also elevated, indicating that these cells are transcriptionally competent.

When comparing the growth of aerobic control cells with post-hypoxic cells, Table II shows that the primary affect is on the lag time. When the post-hypoxic group is separated into two fractions based on size, the smaller cells behave in a similar manner to the unseparated population, but the large cells show an increase in doubling time as well as lag time. The large cells quickly lost most if not all of their extra DNA within the first cell division, indicating that this extra DNA is not stable. However, small amounts of DNA could have been retained (e.g. gene amplification) and would not have been detected using the flow cytometer.

**Figure 8** DNA content of large post-hypoxic cells versus regrowth time. Aerobic cells (A) and cells allowed to regrow 0, 1 and 7 days are shown.

**Table III** Survival of aerobic, post-hypoxic, small post-hypoxic, and large post-hypoxic cells

| *Sample* | *PE*[a] | *CAIS*[b] |
|---|---|---|
| Aerobic | 1.00 | 1.04±0.11 |
| Post-hypoxic | 0.63±0.04 | 0.60±0.06 |
| Smaller | 0.82±0.06 | 0.92±0.07 |
| Larger | 0.13±0.03 | 0.11±0.06 |

[a]Survival measured by colony forming capacity (plating efficiency). Errors = ±s.e.m. of 7–9 experiments. [b]Survival measured using the cell analyser imaging system. Errors = ± s.e.m. of 3 experiments.

**Table IV** CAIS survivals from Table III subdivided into small and large fractions based on cell analyser parameters

| *Sample* | *CAIS*[a] | *Smaller*[b] | *Larger*[c] | *%large*[d] |
|---|---|---|---|---|
| Aerobic | 1.04±0.11[e] | 1.05±0.12 | 0.97±0.07 | 16± 7 |
| Post-hypoxic | 0.60±0.06 | 0.74±0.17 | 0.48±0.14 | 50±37 |
| Smaller | 0.92±0.07 | 0.92±0.05 | 0.75±0.12 | 10± 7 |
| Larger | 0.11±0.06 | 0.35±0.06 | 0.10±0.05 | 94± 5 |

[a]Overall survival as determined using the cell anayser. [b]Survival of the smaller cells in each sample. [c]Survival of the larger cells in each sample. [d]Percentage of larger cells in each sample. [e]Errors = ±s.e.m. of 3 experiments.

Clonogenic survival of cells exposed to 20 h of hypoxia and allowed to recover for 15 h was found to be 63%. This agrees with other results from this laboratory indicating survival of slightly greater than 50% for cells exposed to 24 h of hypoxia (Wilson *et al.*, 1986), with very little recovery of survival following re-aeration. When these cells were separated into two cell populations, those that were larger than normal control cells had a survival of 13%, significantly less than that of the unseparated population. This decrease in clonogenicity is also reflected in the doubling time of these cells when replated. It was considered possible that the large cells were reproductively dead cells, and that the survival seen was due to contamination from smaller cells. Others have shown that these large cells have over-replicated DNA and undergo gene amplification resulting in acquired drug resistance (Rice *et al.*, 1985, 1986) and enhanced metastatic potential (Young *et al.*, 1988). For this to be possible, at least some of the large cells must be clonogenic.

The cell analyser potentially provides a means to determine whether the clonogenic cells in the large cell fraction arise from actual large cells and not from contaminating smaller cells. By using the analyser to locate cells intially and to then follow them and measure colony formation, one can ascertain the characteristics of the initial cells that were clonogenic. This technique allowed us to analyse separately our large and small cell populations obtained earlier. We found that in all populations the smaller cells had a higher survival than the larger cells, but the larger cells did have some reproductive capacity. Visual inspection of the individual plated cells and their resulting colonies confirmed that some of the larger cells were clonogenic.

When synchronised cells were exposed to hypoxia and allowed to recover, the greatest degree of large cell formation and DNA overproduction was found among the cell populations consisting of largely late $G_1$ and S phase cells before

**Figure 9** 'Large' cells originate from S phase. Cells were separated into six fractions using centrifugal elutriation. The horizontal axis of all six curves represents the six fractions. The top half of the figure represents the DNA distributions of these fractions. The vertical axis represents the percentage of cells in each cell cycle phase with $G_1 + S + G_2\text{-}M = 100\%$ for each fraction. All six fractions were then exposed to 20 h of hypoxia and allowed to recover for 15 h. The lower half of the figure represents the proportion of cells in each fraction containing increased amounts of DNA, RNA, or increased size when compared to aerobic control cells. Maximum percentages of cells with enhanced DNA was 61% (fraction 3) and enhanced RNA was 65% (fraction 3). Almost all the cells in fraction 3 showed increased size.

hypoxia. This is consistent with the hypothesis of Schimke that gene amplification occurs in cells intially halted in S phase (Rice *et al.*, 1986; Schimke, 1985; Schimke *et al.*, 1985).

When the large cells are re-plated, the average size of the population decreases exponentially, reaching the size of aerobic control cells by 90 h. Most of the loss in size occurs as the large cells divide, forming daughter cells that are closer in size to normal exponential cells. DNA histograms obtained during this regrowth period also show a rapid return to apparantly normal DNA levels. The first cell division produces predominantly $G_1$ cells of normal 2C DNA content. Since the flow cytometer divides the S phase region of the DNA histogram into only about 100 channels, it is unable to detect amplification of a small number of individual genes, all that would be necessary for development of drug resistance. While it is clear from our data that almost all of the overproduced DNA present in the large cells is lost quickly, it is still possible that enough remains to produce altered cell function.

Elsewhere, we have reported the development of significant resistance of cells to adriamycin, when exposed to the drug in air, immediately following severe hypoxia (Wilson *et al.*, 1989). This resistance declined with increasing aerobic recovery time after hypoxia. By 15 h of recovery (following 20 h hypoxia) only a small degree of adriamycin resistance remained. As described above, these conditions result in the development of a population of cells with markedly overproduced DNA (large cells). These large cells were no more resistant to adriamycin than the small cells with normal DNA content (Wilson *et al.*, 1989). Rice *et al.* (1986) have reported the presence of methotrexate resistance in cells with overproduced DNA, but not in cells with normal DNA content. Apparently greatly overproduced DNA is neither necessary nor sufficient for the development resistance to certain drugs, but hypoxia itself can generate significant resistance to a subsequent adriamycin exposure.

While hypoxia has sudden effects on cell growth, recovery from hypoxia produced more gradual changes. The severeity of the changes is directly related to the duration of the hypoxic stress. The cell cycle is grossly perturbed during recovery with initial blocks preventing cells from entering or leaving $G_2$-M phase and the accumulation of cells in $G_2$-M phase, some with increased size and greater than 4C DNA content. As a population, these large cells show impaired cell growth and survival, and quickly lose most of their extra DNA. The origin of these cells is consistant with current hypotheses about gene amplification. The stable incorporation of even a small fraction of this extra DNA can possibly have significant effects on cell function.

As discussed above, hypoxia and recovery from hypoxia produce many changes in cells *in vitro*. Whether similar events occur *in vivo* is unknown. Hypoxic cells have been demonstrated in tumours (Powers & Tolmach, 1963; Wendling *et al.*, 1985), as well as the phenomenon of reoxygenation (Rockwell & Moulder, 1985; van Putten & Kallman, 1968). *In vivo* systems are influenced by many factors, of which hypoxia is just one (Sutherland *et al.*, 1986). Care must be taken in extrapolating these results to such systems, but these results show that hypoxia and recovery from hypoxia can have potentially large effects cell growth and macromolecular synthesis.

All flow cytometry and centifugal elutriation were performed using the flow cytometry and cell separation facilities of the University of Rochester Medical Center Cancer Center. We would like to thank Brenda King and Snow Bui of their expert help with centrifugal elutriation and flow cytometry. This work was supported by ACS grant PDT-320 and NIH Medical Scientists Training Program grant 5-T32-GM07356.

### References

BAGWELL, C.B. (1979). Theory and application of DNA histogram analysis. PhD thesis, University of Miami, Coral Gables, Florida.

BORN, R. & EICHOLTZ-WIRTH, H. (1981). Effect of different physiological conditions on the action of adriamycin on chinese hamster cells *in vitro*. *Br. J. Cancer*, **44**, 241.

DEAN, P.N. & JETT, J.H. (1974). Mathematical analysis of DNA distributions derived from flow microfluorometry. *J. Cell Biol.*, **60**, 523.

HEACOCK, C.S. & SUTHERLAND, R.M. (1986). Induction characteristics of oxygen regulated proteins. *Int. J. Radiat. Oncol. Biol. Phys.*, **12**, 1287.

KENG, P.C., LI, C.K.N. & WHEELER, K.T. (1980). Synchronization of 9L rat brain tumor cells by centrifugal elutriation. *Cell Biophys.*, **2**, 191.

KOCH, C.J., KRUUV, J. & FREY, H.E. (1973). Variation in radiation response of mammalian cells as a function of oxygen tension. *Radiat. Res.*, **53**, 33.

LUK, C.K., KENG, P.C. & SUTHERLAND, R.M. (1985). Regrowth and radiation sensitivity of quiescent cells isolated from EMT6/Ro fed plateau monolayers. *Cancer Res.*, **45**, 1020.

MARTIN, W.M.C. & MCNALLY, N.J. (1980). Cytotoxicity of adriamycin to tumour cells *in vivo* and *in vitro*. *Br. J. Cancer*, **42**, 881.

PALCIC, B. & JAGGI, B. (1986). The use of solid-state image sensor technology to detect and characterize live mammalian cells growing in tissue culture. *Int. J. Radiat. Biol.*, **50**, 345.

POWERS, W.E. & TOLMACH, L.J. (1963). A multicomponent x-ray survival curve for mouse lymphosarcoma cells irradiated *in vivo*. *Nature*, **197**, 710.

RICE, G.C., HOY, C. & SCHIMKE, R.T. (1986). Transient hypoxia enhances the frequency of dihydrofolate reductase gene amplification in chinese hamster ovary cells. *Proc. Natl Acad. Sci. USA*, **83**, 5978.

RICE, G.C., SPIRO, I.J. & LING, C.C. (1985). Detection of S-phase overreplication following chronic hypoxia using a monoclonal anti-BrdUrd. *Int. J. Radiat. Oncol. Biol. Phys.*, **11**, 1817.

ROCKWELL, S. & MOULDER, J.E. (1985). Biological factors of importance in split-course radiotherapy. In *Optimization of Cancer Radiotherapy*, Paliwal, B.R., Herbert, D.E. & Orton, C.G. (eds) p. 171. American Institute of Physics: New York.

SCHIMKE, R.T. (1985). Methotrexate resistance and gene amplification. *Cancer*, **57**, 1912.

SCHIMKE, R.T., HILL, A. & JOHNSTON, R.N. (1985). Methotrexate resistance and gene amplification: an experimental model for the generation of cellular heterogeneity. *Br. J. Cancer*, **51**, 459.

SHRIEVE, D.C., DEEN, D.D. & HARRIS, J.W. (1983). Effects of extreme hypoxia on the growth and viability of EMT6/SF mouse tumor cells *in vitro*. *Cancer Res.*, **43**, 3521.

SMITH, E., STRATFORD, I.J. & ADAMS, G.E. (1980). Cytotoxicity of adriamycin on aerobic and hypoxic chinese hamster V79 cells *in vitro*. *Br. J. Cancer*, **41**, 568.

SUTHERLAND, R.M., FREYER, J., MUELLER-KLIESER, W. & 4 others (1986). Cellular growth and metabolic adaptation to nutrient stress environment in tumor microregions. *Int. J. Radiat. Oncol. Biol. Phys.*, **12**, 611.

SUTHERLAND, R.M., KENG, P.C., CONROY, P.J., MCDERMOTT, D., BAREHAM, B.J. & PASSALACQUA, W. (1982). *In vitro* hypoxic cytotoxicity of nitroimidazoles: uptake and cell cycle phase specificity. *Int. J. Radiat. Oncol. Biol. Phys.*, **8**, 745.

TANNOCK, I.F. (1972). Oxygen diffusion and the distribution of cellular radiosensitivity in tumours. *Br. J. Radiol.*, **45**, 515.

VAN PUTTEN, L.M. & KALLMAN, R.F. (1986). Oxygen status of a transplantable tumor during fractionated radiotherapy. *J. Natl Cancer Inst.*, **40**, 441.

WENDLING, P., MANZ, R., THEWS, G. & VAUPEL, P. (1985). Heterogenous oxygenation of rectal carcinomas in humans: a critical parameter for preoperative radiation? In *Oxygen Transport to Tissue VI*, Bruley, D., Bicher, H. Raneau, D. (eds) p. 293. Plenum Publishing: New York.

WILSON, R.E., BAUER, K.D. & SUTHERLAND, R.M. (1984). A multidimensional computer program for cell cycle analysis. In Proceedings, Analytical Cytology X, Pacific Grove, CA. P. D52.

WILSON, R.E., KENG, P.C. & SUTHERLAND, R.M. (1986). Quantitation and kinetics of oxygen regulated protein induction. Abstracts of Papers for the Thirty-Fourth Annual Meeting of the Radiation Research Society. Abstract En-20.

WILSON, R.E., KENG, P.C. & SUTHERLAND, R.M. (1989). Drug resistance in Chinese hamster ovary cells during recovery from severe hypoxia. *J. Natl Cancer Inst.*, **81**, 1235.

WILSON, R.E. & SUTHERLAND, R.M. (1989). Enhanced synthesis of specific proteins, RNA, and DNA caused by hypoxia and reoxygenation. *Int. J. Radiat. Oncol. Biol. Phys.*, **16**, 957.

YOUNG, S.D., MARSHALL, R.S. & HILL. R.P. (1988). Hypoxia induces DNA overreplication and enhances metastatic potential of murine tumor cells. *Proc. Natl Acad. Sci. USA*, **85**, 9533.